# A Versatile, Incubator‐Compatible, Monolithic GaN Photonic Chipscope for Label‐Free Monitoring of Live Cell Activities

**DOI:** 10.1002/advs.202200910

**Published:** 2022-04-11

**Authors:** Yong Hou, Jixiang Jing, Yumeng Luo, Feng Xu, Wenyan Xie, Linjie Ma, Xingyu Xia, Qiang Wei, Yuan Lin, Kwai Hei Li, Zhiqin Chu

**Affiliations:** ^1^ Department of Electrical and Electronic Engineering The University of Hong Kong Hong Kong China; ^2^ School of Microelectronics Southern University of Science and Technology Shenzhen 518055 China; ^3^ Department of Biotherapy State Key Laboratory of Biotherapy and Cancer Center West China Hospital Sichuan University Chengdu Sichuan 610065 China; ^4^ Department of Mechanical Engineering The University of Hong Kong Hong Kong China; ^5^ College of Polymer Science and Engineering State Key Laboratory of Polymer Materials and Engineering Sichuan University Chengdu 610065 China; ^6^ Advanced Biomedical Instrumentation Centre Hong Kong Science Park Shatin New Territories Hong Kong; ^7^ School of Biomedical Sciences The University of Hong Kong Hong Kong China

**Keywords:** chipscope, gallium nitride (GaN), label‐free sensing, living cell activities, monolithic integration

## Abstract

The ability to quantitatively monitor various cellular activities is critical for understanding their biological functions and the therapeutic response of cells to drugs. Unfortunately, existing approaches such as fluorescent staining and impedance‐based methods are often hindered by their multiple time‐consuming preparation steps, sophisticated labeling procedures, and complicated apparatus. The cost‐effective, monolithic gallium nitride (GaN) photonic chip has been demonstrated as an ultrasensitive and ultracompact optical refractometer in a previous work, but it has never been applied to cell studies. Here, for the first time, the so‐called GaN chipscope is proposed to quantitatively monitor the progression of different intracellular processes in a label‐free manner. Specifically, the GaN‐based monolithic chip enables not only a photoelectric readout of cellular/subcellular refractive index changes but also the direct imaging of cellular/subcellular ultrastructural features using a customized differential interference contrast (DIC) microscope. The miniaturized chipscope adopts an ultracompact design, which can be readily mounted with conventional cell culture dishes and placed inside standard cell incubators for real‐time observation of cell activities. As a proof‐of‐concept demonstration, its applications are explored in 1) cell adhesion dynamics monitoring, 2) drug screening, and 3) cell differentiation studies, highlighting its potential in broad fundamental cell biology studies as well as in clinical applications.

## Graphical abstract

A low‐cost, highly integrated, and incubator‐compatible GaN‐based refractive index‐sensitive chipscope has been developed for living cell activities monitoring. It enables not only photoelectric readout of cellular/subcellular refractive index changes, but also direct imaging of cellular/subcellular morphology in real‐time.

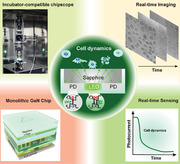

## 1 Introduction

Moving beyond the mere “snapshot” provided by conventional endpoint assays (e.g., colorimetry), live cell sensing technologies have become more popular recently in biosensor development due to their ability to achieve real‐time monitoring of biological processes such as adhesion, proliferation, and apoptosis.^[^ ^1^, ^2^ ^]^ This ability may eventually lead to important new applications in drug discovery, cell invasion and migration monitoring, and toxicity detection. In particular, the rapidly advancing biotechnology industry has called for sensors with features such as miniaturization, intellectualization, expansibility, multifunctionalization and low cost.^[^ ^3^, ^4^ ^]^

To date, many sensing approaches with label‐free properties have been proposed. The most widely used one is the electric cell‐substrate impedance method, which has been commercialized on the market.^[^ ^5^ ^]^ Typically, this kind of nonoptical sensor contains an array of gold biosensors integrated into the well plate so that real‐time impedance measurement can be conducted to track adhesion‐related dynamics in cells.^[^ ^6^ ^]^ However, the electrical stresses induced by the exposed electrode can affect cell viability, thus limiting the method's application in many situations.^[^ ^7^ ^]^ As alternatives, optical approaches, including resonant waveguide grating biosensor (RWG) and surface plasmon resonance (SPR), have attracted intensive interest in recent years due to their no‐invasive and label‐free nature.^[^ ^8^, ^9^, ^10^ ^]^ Both RWG and SPR employ a surface‐bound optical evanescence field with a decay (penetration) length of hundreds of nanometers to probe changes in the refractive index (RI) of cells caused by, for example, their adhesion and spreading.^[^ ^11^, ^12^ ^]^ Specifically, RWG sensors monitor the shift in resonance wavelength or angle by using sophisticated optics (spectrometer) and electronics,^[^ ^13^ ^]^ making them difficult to integrate into a low‐cost and miniature device for widespread use. SPR technology utilizes a prism to induce plasmon resonance on a patterned thin metal surface with a fixed angle of incident laser beam. To enhance the sensing capability, additional angle and wavelength scanning units have to be integrated into the system to widen dynamic detection range and increase refractive index (RI) resolution.^[^ ^14^, ^15^ ^]^

In recent years, by utilizing interactions between biomarkers and specifically designed nano/microstructures, optical chip‐based devices have emerged as a promising technique for realizing low‐cost and high‐sensitivity biosensing.^[^ ^16^, ^17^ ^]^ In particular, the high miniaturization, intellectualization and expansibility of such devices enable them to meet various challenges in practical applications. Among the different materials suitable for constructing optoelectronic devices, GaN semiconductor and its alloys are widely used because of their inherent long‐life span, high efficiency, and excellent physical and chemical stability.^[^ ^18^ ^]^ They can also radiate and absorb high‐energy photons, making them ideal for fabricating light‐emitting diodes (LEDs) and photodetectors (PDs) that operate in the blue, green, and even ultraviolet spectral ranges.^[^ ^19^ ^]^ Following this line of reasoning, our group has recently developed a series of GaN‐based, chip‐scale optical sensors for environmental (humidity, pressure, salinity) monitoring.^[^ ^18^, ^20^, ^21^ ^]^ In particular, we have demonstrated that a monolithic integration scheme can be utilized to form InGaN/GaN LEDs and PDs in the same microchip. As there is no need for external optics and costly spectrum analyzers, this highly miniaturized LED‐PD configuration greatly simplifies the sensor setup and therefore makes it possible to achieve mass production and easy integration with other platforms.

In the present paper we describe our development of a low‐cost, highly integrated, and incubator‐compatible GaN‐based RI chipscope for label‐free monitoring of cellular activities. Specifically, the chip incorporating a mini‐DIC microscope allows us not only to perform real‐time photocurrent measurement (and hence track changes in cell morphology, motions and cell‐cell interactions), but also to collect brightfield live‐cell images simultaneously. Utilizing this chipscope, we successfully tracked the adhesion‐spreading‐detaching dynamics of cells. Our device was also capable of capturing drug‐induced cancer cell apoptosis and immune cell differentiation, demonstrating its potential for use in practical biosensing applications.

## 2 Results and Discussion

### 2.1 Design of GaN Chipscope for Sensing and Imaging

**Figure 1:**
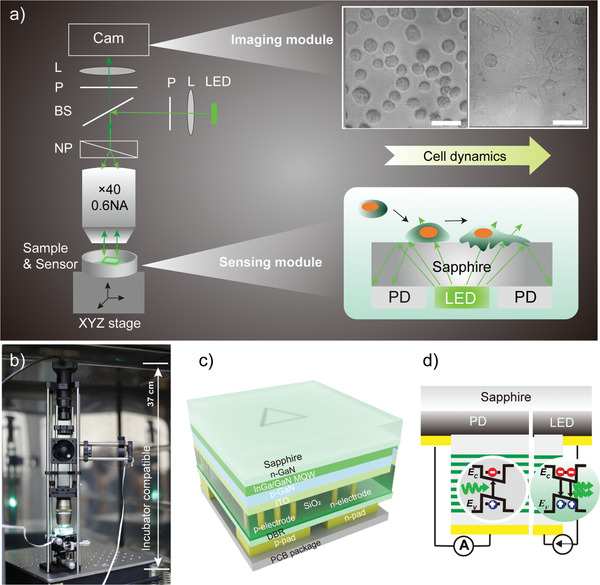
The working principle of the GaN chipscope. a) The optical setup of the monolithic GaN photonic chipscope. Cam: camera, L: lens, P: polarizer, BS: beam splitter, NP: Normarski prism. The insert (upper) shows the optical image of the cells obtained from the mini‐DIC scope. The insert (bottom) indicates the mechanism of sensing: the chip works as a refractometer for measuring living cell activities associated with RI changes. Scale bar indicates 50 µm. b) Optical images of the GaN chipscope inside the cell incubator. c) Schematic composition of the adopted InGaN‐based monolithic photonic chip. d) Schematic diagram depicting the different mechanisms of emission and detection using the same quantum structure.

Real‐time monitoring of the activities of living cells and their therapeutic responses is vital for applications such as disease diagnosis and pharmacodynamic analysis. Here, we propose an integrated miniature sensing and imaging system to achieve this. Specifically, the system consists of two core components: i) a monolithic optoelectronic chip; and ii) a mini‐DIC microscopy unit (**Figure** **1**a,b). The former integrated LED and PD on a GaN/sapphire chip through wafer‐scale processes (see the Experimental Section). Importantly, the LED and PD parts were electrically isolated to each other, but can work independently by connecting to a current source and an ammeter, respectively, as shown in Figure 1d. In this sense, a triangular LED with a side length of 238 µm was located at the center of the chip, whereas the surroundings corresponded to the light‐detecting region, as shown in Figure 1c and Figure S1a (Supporting Information). The DIC unit used a prism to split the linearly polarized light into two rays which experienced different optical paths due to the varied thickness of the specimen. Hence, the light beams with different phases caused by optical path differences underwent interference and generate amplitude fluctuations to form the DIC images (Figure 1a). The exposed sapphire substrate is favorable in direct contact with cells, as shown in Figure 1a. Several such miniature systems were fabricated on a 1×1 mm^2^ chip, bonded on a printed circuit board , to connect with a current source and ammeter easily. Additionally, to ensure that cells could be cultured on the chip, we fabricated a mini‐polydimethylsiloxane (PDMS) chamber (1×1 cm^2^) to enclose the chip (Figure S1, Supporting Information). Importantly, the dimensions of the device were only 7 × 17 × 37 cm, allowing it to be placed and work in a cell incubator (Figure 1b). Detailed setup descriptions are provided in Methods.

### 2.2 Sensing Principle of the Optoelectronic Chip

As a monolithic integration design of the chip, the same shared sapphire substrate can realize the light coupling from the LED to the PD without any external optics, as shown in Figure 1a. In particular, when the lights emitted from LED reached external media with a low RI (i.e., *n*_media_ < *n*_sapphire_), total internal reflection occurred at the sapphire/media interface, causing some light to be reflected into the light‐detecting region. The intensity of the reflected light was then detected by the PD. Since the critical angle enlarges as the refractive index increases, less light will be reflected back to the PD, resulting in a decrease in the photocurrent. When the cells are in contact with the chip surface, the sensor provides a rapid photocurrent response due to the obvious RI change from sapphire‐culture medium (1.78–1.337) to sapphire‐cells (1.78 to 1.343–1.48).^[^ ^22^, ^23^ ^]^ Furthermore, the formation of strong cell‐substrate adhesion and/or the evolution of cell morphology could also result in changes in the recorded photocurrent. This will be discussed later.

### 2.3 Demonstration of Optical and Electrical Performance and Sensing Ability

**Figure 2:**
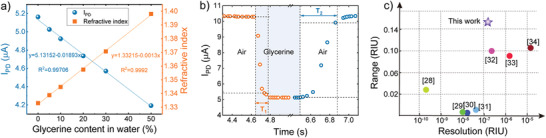
Validating the sensing capability of the GaN chip in the model system. a) Plot of the measured refractive index using the Abbe refractometer (orange square) and photocurrent response of the sensing device (blue circle) under glycerin contents ranging from 0% to 50%. b) The instantaneous response of the GaN chip. c) Comparison with other reported work in sensing the refractive index of mediums.^[^ ^28^, ^29^, ^30^, ^31^, ^32^, ^33^, ^34^ ^]^

Before applying this chip device for monitoring cellular behaviors, some basic electrical characteristics of the on‐chip LED and PD were conducted (see Figure S2, Supporting Information). We also used glycerin/water mixtures to test the sensing sensitivity, response speed, and stability of the chip device. **Figure** **2**a shows the detected photocurrent as a function of glycerin concentration, varying from 0% to 50% and therefore altering the medium RI.^[^ ^24^ ^]^ Extracted from the fitted linear slope, the sensitivity of the sensor was found to be around 18.93 nA/% (or 149 216 nA/RIU). The sensing resolution of the chip device was found to be 2.641×10^–3^%, which was determined by the resolution of the Keithley 2450 ammeter of 0.05 nA. Next, the chip response speed was quantified by a cycle test by switching the testing mediums between glycerin solution and air. The chip device responded very quickly showing only 0.169 s for the decline time T1 (from air to glycerin) and 0.386 s for the rise time T2 (from glycerin to air) (Figure 2b). The rapid response time was mainly contributed to the fast photon‐electron conversion property of the chip device incorporating InGaN/GaN MQWs (Figure S3, Supporting Information).^[^ ^25^, ^26^, ^27^ ^]^ Additionally, compared with reported methods in sensing media refractive index, this sensor exhibited a comparable sensing resolution but a much larger sensing range (RI: 1.333‐1.48) (Figure 2c). Herein, the theoretical sensing range will be larger (up to 1.78 of sapphire) based on the working principle of this sensor (see the last section). Lastly, we conducted the simulation by building a sandwich model with sapphire‐intermediate‐sensing layer to characterize the vertical sensing range of the chip in water. The maximum theoretical vertical sensing ranges are 500 and 300 nm in water and air, respectively (Figures S4 and S5, Supporting Information).The demonstration of the chip device in high sensing sensitivity, range, resolution, vertical sensing ranges and rapid response speed demonstrates its potential ability to detect more challengeable cell behaviors.

### 2.4 Assessing the Visible Cellular Dynamics via the Monolithic GaN Photonic Chipscope

**Figure 3:**
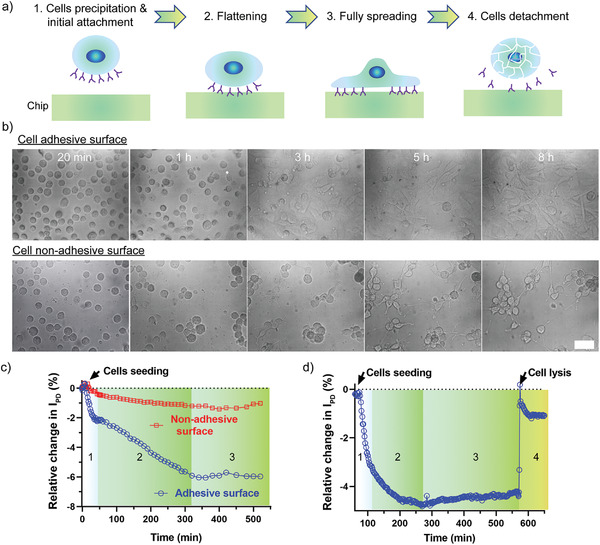
Label‐free monitoring of cell adhesion and detachment via the GaN chipscope. a) Schematic illustration of cell adhesion phases. b) DIC images of 3T3 cells grown on the adhesive (top panel) and nonadhesive (bottom panel) surfaces from time 20 min to 8 h. Scale bar indicates 100 µm. c) Relative photocurrent changes as a function of time for cells on adhesive surface (blue line) and nonadhesive surface (red line), respectively. The photocurrent data collection time interval gradually varied with time (0–1 h: 5 min per point; 1–5 h: 10 min per point; 5–9 h: 20 min per point). d) Relative photocurrent changes as a function of time for cell deposition, initial attachment, spreading, and detachment. The photocurrent data was obtained at a rate of 2 min per point (pause mode, irradiation for 5 s–pause for 115 s–irradiation for 5 s).

As a proof of concept that the GaN chipscope is capable of tracking the activities of living cells, we studied its ability to sense cell adhesion, a process that is critical for the formation of tissues and organs and participates in a large number of physiological and pathological processes, such as cell differentiation, immune response, inflammation, and tumor metastasis.^[^ ^35^, ^36^ ^]^ In general, cell adhesion includes three steps: cell precipitation and initial cell‐substrate contact, cell flattening and full spreading (**Figure** **3**a).^[^ ^37^ ^]^ During these processes, the main observable change is the morphological transition of the cell from being spherical to flat, resulting in a gradual increase in the coverage of cells on the chip surface. This significantly changes the average RI contrast at the cell‐chip interface, thereby altering the photocurrent generated by the PD.

NIH 3T3 cells were used in the present study due to their rapid adhesion response and significant cell area changes during spreading. We first performed a live/dead assay test to evaluate the potential phototoxicity of our chip to the cells. The results showed that cells exposed to green light in both continuous and pulsed modes maintained relatively high viability (>80%) even after 24 h of treatment, indicating that our sensor chip is biocompatible for long‐term cell measurement (Figure S6, Supporting Information). Next, after the chip was stabilized in the incubator at 37 ℃, the cell suspension was added into the chip chamber. Interestingly, the photocurrent dropped sharply by ≈2.17%) in the next 30 min (despite some initial signal fluctuations), then decreased much more slowly for another 4.5 h before becoming saturated (Figure 3c). Benefitting from the integrated mini‐DIC imaging system, we can clearly capture the cell morphology changes in real‐time. As shown in the top panel of Figure 3b and Video S1 (Supporting Information), the round cells gradually precipitated onto the chip surface in the first 30 min (step 1), and then started to extend in the next 4 h (step 2). After that, they continued to flatten and formed a dense monolayer sheet covering the entire chip surface (step 3). These observed cell morphology changes closely matched the optical response of the chip, indicating the successful integration of GaN chip ‐based sensing and DIC imaging units in our system for cellular activity monitoring. Additionally, to mimic the cell detachment process (step 4), we treated the cells with sodium dodecyl sulfate (SDS), a known surfactant that can detach and lyse adhering cells from culture dishes or flasks.^[^ ^38^ ^]^ Unsurprisingly, the cell monolayer became completely detached from the chip surface within a few seconds of SDS loading (figures not shown). Accordingly, the signal showed a rapid increase (4.24%) after the addition of SDS, and then slowly returned to the level corresponding to the photocurrent value before cell seeding (Figure 3d). This demonstrated that our platform could not only capture the instant RI changes at the chip‐medium interface, but also exhibited excellent stability for long‐term measurement.

As a control experiment, we also coated the chip surface with antifouling polymers (see the detailed protocol and Figure S7, Supporting Information) that can effectively prevent cell‐surface adhesion.^[^ ^39^ ^]^ Under such circumstances, cells were found to roll onto (rather than attach to) the chip surface in the first hour. Aggregation of cells took place in the next 8 h, while no cell spreading was observed (Figure 3b and Video S2, Supporting Information). Interestingly, despite a slight decrease of photocurrent by 0.55% in the first 60 min, the signal largely remained constant throughout the experiment (Figure 3c).

### 2.5 Accessing the Invisible Cellular Dynamics via the Monolithic GaN Photonic Chipscope

**Figure 4:**
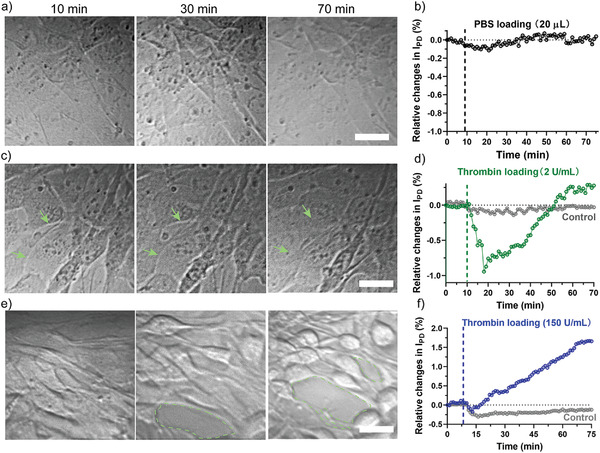
Label‐free monitoring of intracellular dynamics via the GaN chipscope. Photocurrent variations as a function of time of 3T3 cells monolayer and their corresponding DIC images after a,b) stimulation by PBS, c,d) low dose of thrombin (2 U mL^−1^), e,f) and high dose of thrombin (150 U mL^−1^), respectively. The photocurrent data was obtained at a rate of 1 point min^−1^ (pause mode, irradiation for 5 s–pause for 55 s–irradiation for 5 s). Yellow arrows in (c) show the slight morphological changes as a result of low dose thrombin stimulation. Red dots areas in (e) represent the exposed chip surface due to drug‐induced cell shrinkage. Scale bar indicates 30 µm.

To investigate the ability of the GaN chipscope to recognize intracellular dynamics, the chipscope responses under the stimulation of cells with various biomolecules and chemicals were measured and compared in **Figure** **4**. The photocurrent signals with DIC images taken at different time intervals after the stimulation were used to determine the morphological origin of the photocurrent signals variations. As a negative control, PBS was utilized to stimulate the cells to address the possibility of photocurrent changes being solely affected by the interference from the liquid shear force in the chip chamber. As expected, no apparent variations in the photocurrent signal were observed after the PBS was loaded (Figure 4a). Our imaging unit confirmed this result, showing no detectable change in the cell morphology (Figure 4b).

Next, the cells were stimulated with thrombin at two different doses. Thrombin is a serine protease that is well known to be implicated in hemostasis and vascular endothelium permeability. Actually, cells can interact with thrombin through the thrombin receptors, which have been identified on many types of cells, including endothelial cells,^[^ ^40^ ^]^ smooth muscle cells,^[^ ^41^ ^]^ neuronal cells,^[^ ^42^ ^]^ fibroblasts,^[^ ^43^ ^]^ and peripheral blood lymphocytes,^[^ ^44^ ^]^ etc. A low dose of thrombin has been shown to temporarily increase the internal elastic tension by enhancing the activity of the Ca^2+^ based myosin light chain.^[^ ^45^ ^]^ By contrast, a high dose of thrombin is toxic and induces cell death by cleavage of DNA into fragments.^[^ ^46^ ^]^ As shown in Figure 4, after stimulation with a low dose of 2 U mL^−1^ thrombin, the cells showed a biphasic response. In the first 10 min, the signal dramatically decreased (0.94%), probably as a result of the increase in RI induced by the sharp increase in intracellular Ca^2+^ concentration after thrombin (low dose) loading.^[^ ^43^ ^]^ In the next 30 min, the signal returned to its initial level. Since the working time of thrombin is within a range of minutes, the local concentration of Ca^2+^ gradually returned to the normal state, resulting in a recovery phase of photocurrent in the following 30 min. The result was further confirmed by a living cell calcium tracking experiment, where a similar biphasic cell response was observed in the presence of low‐dose thrombin. This result perfectly matched the data recorded by our GaN chipscope (Figure S8 and Video S3, Supporting Information). Importantly, the cell spreading area and cell morphology monitored by the imaging system showed no visible changes, and there was only slight cell extension after thrombin treatment (yellow arrows labeled, Figure 4c), indicating that the intracellular dynamics dominate the photocurrent generation.

We then increased the dose of thrombin to 150 U mL^−1^ and observed a rapid increase of photocurrent throughout the tested period. The photocurrent reached a plateau after 50 min and then stabilized, with a maximal change of 1.66% (Figure 4f). Consistent with the photocurrent data, the imaging unit monitored an increase in the intercellular space due to cell shrinkage induced by thrombin (Figure 4e). In this case, the cell spreading area and optical current data were negatively correlated with each other. To corroborate this result, blebbistatin was used to induce the cell morphology changes by inhibiting the myosin II activity. Myosin II is a critical determinant of contractile characteristics of cell motility and cell adhesion in several tissue types. After treatment with the drugs (50 × 10^−6^ m), the cells gradually shrank in the next 1 h, showing increased cell gaps and dendritic cell morphologies (Figure S9a, Supporting Information). As expected, an increase in photocurrent was observed during this period due to the decrease of the cell coverage area on the chip surface (Figure S9b, Supporting Information).

Cells can respond to chemical stimuli in a variety of ways, including the activation of signaling pathways, morphological changes, and the initiation of cell death.^[^ ^46^, ^47^ ^]^ The currently available systems on the market for label‐free and real‐time monitoring of cells in vitro are mostly based on cell morphology/cell spreading, which means that the cell activities can only be measured when there are cell morphological changes or cell spreading variations. In this study, our newly developed GaN chip was able not only to measure changes in cell morphology/cell spreading, but also to sense and record RI dynamics induced by intracellular dynamics in real‐time. It was even capable of determining the dominant factor contributing to the RI changes with the help of the imaging analysis. These unique features make the GaN chipscope an excellent candidate for monitoring cell response against various drugs and chemicals in vitro in a variety of ways.

### 2.6 GaN Chipscope in the Demonstration of Drug Screening

**Figure 5:**
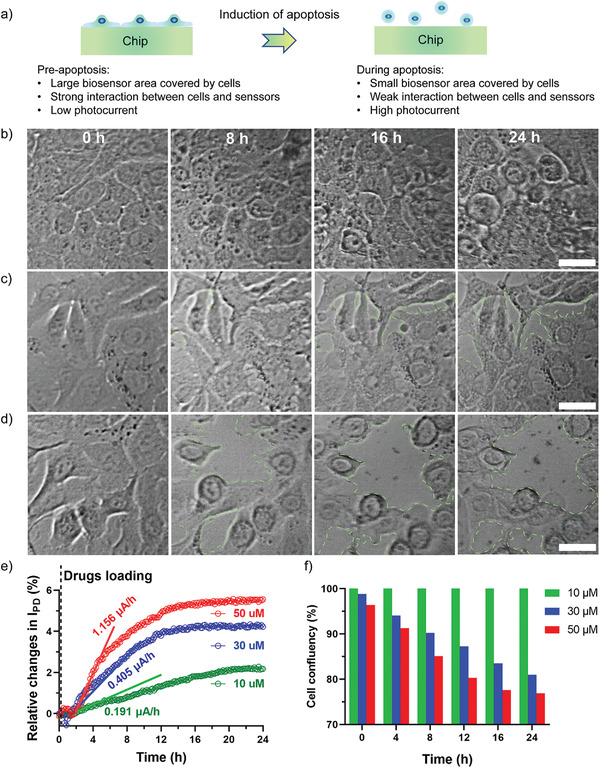
GaN chipscope in the application of drug‐cell interactions. a) Schematic illustration of drug‐induced cell apoptosis. Representative images of A549 cells treated with anticancer drugs with varied concentrations at specific time points b) 10 × 10^−6^ m, c) 30 × 10^−6^ m, d) 50 × 10^−6^ m. e) Photocurrent variations as a function of incubation time (A549 cell monolayers treated with drugs with varying concentrations). The solid lines represent the liner fitting to the data. The photocurrent data were obtained at a rate of 10 min per point (pause mode, irradiation for 5 s–pause for 595 s–irradiation for 5 s). f) The cell confluency variations as a function of the drug stimulation time and dose.

To demonstrate the potential application of this sensing platform in drug research, an experiment was conducted to determine the cytotoxicity of the anticancer drug *β*‐lapachone on human lung adenocarcinoma cells (A549).^[^ ^48^ ^]^ The A549 cells were seeded in the chamber of the platform and cultured for 24 h for fully spreading. Afterward, 10 × 10^−6^ , 30 × 10^−6^ or 50 × 10^−6^ m of *β*‐lapachone was added into the chambers, respectively. As shown in **Figure** **5**, the chipscope recorded the responses of A549 cells stimulated by *β*‐lapachone. Clearly, the *β*‐lapachone induced dose‐depend cytotoxicity in A549 cells, as characterized by a significant increase in the photocurrent data with increasing drug concentration.

Additionally, the photocurrent curves offered valuable practical information for the study of drug‐cell interactions. First, by plotting the slope of the tangent line, the speed of cell response at different periods was analyzed. For instance, in the first 5 h of stimulation, the response exhibited by the cells at a high *β*‐lapachone concentration (50 × 10^−6^ m) was 6.05 and 2.85 times higher than that at low (10 × 10^−6^ m) and intermediate concentrations (30 × 10^−6^ m), respectively (Figure 5e). Second, the photocurrent curves revealed the reaction time of the cells under different drug concentrations. For instance, A549 cells exhibited a much faster response against a high dose of *β*‐lapachone (12.5 h and 11.6 h for 50 × 10^−6^ m and 30 × 10^−6^ m of *β*‐lapachone, respectively) than against a low dose (20.2 h for 10 × 10^−6^ m of *β*‐lapachone). Indeed, a higher dose is more toxic and induces faster cell death. Third, by coupling the imaging system, we were able to qualitatively and quantitatively analyze the cell–drug interactions. As shown in Figure 5b–d, the cells shrank in a dose‐dependent manner after *β*‐lapachone treatment. Specifically, cells treated with a lower dose (10 × 10^−6^ m) slowly shrank over time, but there was no obvious increase in intercellular spaces during the 24 h. This suggested that the photocurrent dynamics were mainly to be ascribed to the intracellular RI changes induced by the drugs instead of to the changes in the cell spreading area. The A549 cells treated with higher dose of *β*‐lapachone shrank intensely and showed significant intercellular gaps (red dots line labeled) in the intermediate and high dose groups (Figure 5c,d). We believe that the increase in photocurrent in higher dose‐treated cells was contributed by both cell morphology changes as well as by cell intracellular dynamics alternation. Further, the cell confluency was calculated based on the photos taken by the imaging system. As a result, the confluence of cells treated with high, medium, and low doses of *β*‐lapachone decreased by 23.1%, 19.0%, and 0%, respectively after 24 h incubation.

Therefore, this GaN chipscope platform is capable of recording cell response in regard to both cell adhesions and intra‐/intercellular dynamics after drug treatment, demonstrating its practicality as a toxicity biosensor in rapid drug screening studies.

### 2.7 Demonstration of Cell Differentiation Monitoring

**Figure 6:**
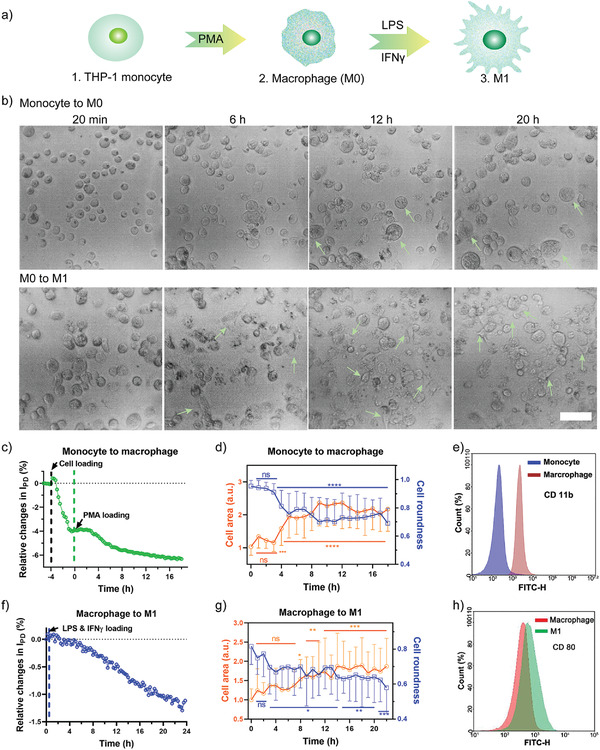
GaN chipscope platform applied in cell differentiation monitoring. a) Schematic illustration of differentiation of THP‐1 monocyte to macrophage with different phenotypes. b) Representative images of monocyte differentiate to M0 cells (upper panel) and M0 cells differentiate to M1 cells (bottom panel), respectively. Scale bar indicates 100 µm. c,d) The relative changes of optical current, cell area, and cell roundness as a function of time during monocyte to M0 differentiation. (*n* = 20–30). e) The specific macrophage surface marker CD 11b expression is illustrated by flow cytometry analysis. f,g) The relative changes of optical current, cell area, and cell roundness as a function of time during M0 to M1 differentiation (*n* = 20–30). h) The specific M1 surface marker CD 80 expression is illustrated by FACS analysis. The photocurrent data was obtained at a rate of 15 min per point (pause mode, irradiation for 5 s ‐pause for 895 s ‐irradiation for 5 s). Cell images were analyzed using Image J (NIH). For quantification of cell spreading area, the shape factor and fluorescent intensity of each cell was readily obtained from Image J measurement.. Data are presented as the mean ± SD, *n* = 20–30. All data were compared with control group at the time 0. *p* values < 0.05 were considered statistically significant (*p < 0.05, **p < 0.01, ***p < 0.001).

The cell refractive index is an intrinsic optical parameter that varies with different cell phenotypes.^[^ ^23^, ^49^ ^]^ This inspired us to explore whether our sensing platform could track online the dynamics of cell differentiation and distinguish the different cell phenotypes. In this experiment, human monocytic THP‐1 cells were employed as the cell model due to their multiphenotypic characteristics, including the initial suspended monocyte, adhered macrophage (M0), and two major polarized states (adhered M1 and M2).^[^ ^50^ ^]^ Here, THP‐1 cells differentiated from suspended state (monocyte) to adhered M0 state by phorbol 12‐myristate 13‐acetate (PMA), followed by induction of the resultant M0 cells to polarize to M1 by LPS/IFN‐gamma. The process was monitored throughout by our GaN chipscope system (**Figure** **6**a).

The photocurrent signal slightly decreased after monocytes were seeded into the chip chamber, indicating that these cells precipitated onto the chip surface due to gravity (Figure 6c). Consistent with the photocurrent signal, the imaging system recorded a fast cell rolling behavior in the initial 30 min before the cells settled down (Figure 6b and Video S4, Supporting Information). PMA (25 ng mL^−1^) was then loaded to trigger monocyte to macrophage differentiation. It is evident from the photocurrent signal that monocytes responded strongly against PMA in 3–9 h after stimulation, and this response became mild in the following 11 h (Figure 6b and Video S4, Supporting Information).

The cell spreading area was calculated according to the photos taken by the imaging system. It was slightly increased in the first 3 h, followed by a significant increase (137%) in cell area in 3–9 h, but fluctuated in the following 11 h. In contrast, the cell roundness exhibited a trend opposite to the cell area (Figure 6d). It was noticed that the imaging data perfectly matched the photocurrent dynamics during the first 9 h after PMA stimulation, which indicated that the photocurrent signals were mainly contributed by the adhesion‐based cell area changes and intracellular dynamic. In the following 10–20 h, the photocurrent data kept decreasing, but the cell area showed no noticeable change, indicating that the cell‐intrinsic property dynamics dominated the signals in this period. To confirm the differentiation of monocyte to macrophage, CD11b as a macrophage surface marker was evaluated by flow cytometry and immunofluorescence staining. After incubation with PMA, the expression of CD11b of THP‐1 cells was significantly increased (Figure 6e), indicating the successful differentiation of monocyte to M0.

To polarize M0 to M1, macrophages were treated with lipopolysaccharide (LPS, 100 ng mL^−1^) and interferon‐gamma proteins (IFN*γ*, 20 ng mL^−1^). A steady decrease of the photocurrent signal was observed in the 24 h following stimulation (Figure 6f). Similarly, the cell spreading area gradually increased by 87% within this 24 h, and the polarized cells showed a “dendritic”‐like morphology with large filopodia (arrows labeled, Figure 6b, Video S5, Supporting Information). By contrast, cell roundness decreased from 0.8 to 0.57 (Figure 6g). There was apparent shifting of M1 macrophage surface maker CD 80, which confirmed the successful differentiation of M0 to M1 macrophage (Figure 6h). Together, these results indicate that our GaN chipscope can monitor in real‐time and quantify cell activities or status changes in both intracellular and intercellular dynamics. Additionally, this device integrated with an imaging system is label‐free and incubator‐adaptive, making it highly suitable for various tests and analyses in living cells in situ.

## 3 Conclusion

Here, we introduce a low‐cost, incubator‐adaptive chipscope based on the refractive index‐sensitive GaN device for label‐free and real‐time cell sensing. The device benefits from its small size, continuous monitoring, and real‐time photocurrent readout and analysis, enabling us to readily capture the dynamics of cell adhesion‐based activities in situ, including cell precipitation, initial attachment, spreading, shrinkage, and detachment. In particular, by coupling the imaging unit and RI sensing unit, the platform can determine both intercellular and intracellular dynamics by monitoring the cell adhesion and morphologies changes with high sensitivity and responsiveness. Another specific outcome of this work is the development of a practical, ready‐to‐use cell analyzer for pharmacological studies to determine the cytotoxicity of anticancer drugs and their corresponding cellular response, as well as cell biology research to track the immune cell phenotypes transform. Technically, these results are sufficiently robust to demonstrate the applicability of the optical chip‐based sensing technology in biosensing.

Compared to the prevailing complex optical living cell biosensing technologies, such as SPR and RWG, our GaN chips tremendously lower the technical thresholds in the design, fabrication and the practical use of biosensors (Table S1, Supporting Information). Specifically, the monolithic strategy was utilized to integrate InGaN/GaN photo emitter and photo detector on the same microchip, which eliminates the use of the costly spectrum analyzer and other optical apparatus. Additionally, due to their microscale size and the less requirement on the sensing setup, the chip can be easily integrated with other devices and applied in some special environments such as fast detection in the wearable device, integration with microscope or working in tight space with high humidity (cell incubator).

Of course, there is room for further improvement in some areas before the device functions optimally as a tool. At present, a major drawback is the limited number of loading samples. For the purpose of technical demonstration, the current chip we used has one sensing unit, only allowing one test and observation online every time. Our lab is presently developing a sensor array which allows the required number/size of sensing modules to fit on one chip (data not shown). This will enable us to perform the high‐throughput single‐cell analysis in the near future. Another limitation is the quality of the imaging system. This can be improved with a more sensitive camera sensor, enabling more details of cell dynamics to be captured.

Assuming that these limitations can be overcome, the GaN chipscope platform has considerable potential as a tool for label‐free monitoring of live cell activities which transcends the boundaries of the conventional “photonic chip” and “microscopy” monitoring processes. The new “chipscope” integrates more functions that highly enrich the data output in both qualitative and quantitative ways. In particular, their easy accessibility and extremely low manufacturing cost (<10 cents per chip) may enable them to be welcomed in the practical use and the market. We believe that our “chipscope” represents an important and exciting advance in the development of biosensors.

## 4 Experimental Section

### Fabrication of the Optoelectronic Chip

The epitaxial structures containing InGaN/GaN multi‐quantum‐wells (MQWs) were grown on a 4 inch sapphire substrate by metal‐organic chemical vapor deposition. The LED and PD mesas were then fabricated on a single wafer by photolithography and inductively coupled plasma (ICP) etching. In order to promote the spreading of current, a 120 nm thick indium‐tin‐oxide (ITO) layer was deposited on the p‐GaN by reactive plasma deposition. The LED and PD were covered by photomasks and a 10 µm wide GaN between them was then ICP‐etched. The p‐electrode and n‐electrode were subsequently patterned by photolithography and then coated with Cr/Al/Ti/Pt/Au materials by electron‐beam evaporation. An insulating SiO_2_ layer with 360 nm thickness was deposited on the wafer by plasma‐enhanced CVD technique. A stacked layer of SiO_2_/TiO_2_ distributed Bragg reflector was deposited as a bottom mirror to reflect the emitted light into the sapphire substrate. The p‐pad and n‐pad regions were defined by photolithography, and a metallization layer was then deposited by electron‐beam evaporation. After rapid thermal annealing, the sapphire substrate was thinned to 150 µm by lapping and polishing process, followed by laser dicing into small chips with the size of 1×1 mm^2^. Both LED and PD possess the same device structure, as shown in Figure 1c.

### Construction of a Mini‐Differential Interference Contrast (DIC) Microscopy

A green GaN chip with an emission wavelength of 520 nm was employed as the light source, and the diffused light beams were further modified through a focal lens. The modulated parallel light propagated through a polarizer and became linearly polarized. After beam splitting, the separated downward beams passing through a birefringent Normarski prism were collected with a 40 × DIC objective with 0.6 NA, and then irradiated on the specimen. The reflected wave fronts experienced varying optical path differences due to irregular specimen surface topography and were gathered by the objective and focused on the interference plane of the prism. The combined lights continued to propagate through the beam splitter and then encountered the analyzer (second polarizer), which allowed the light beams parallel to the analyzer transmission vector to pass through, further undergoing interference and generating amplitude fluctuations at the focal plane of the lens. Finally, the DIC image was captured by a CMOS camera (Thorlabs).

### Cell Culture

NIH 3T3 cells and A549 cells were purchased from ATCC and cultured in DMEM (Gibco) supplemented with 10% bovine growth serum (Gibco) and 1% penicillin/streptomycin (Gibco). NIH 3T3 cells between 6 and 12 passages were used in this study. A549 cells between 4 and 10 passages were used in this study. THP‐1 cells were purchased from ATCC. The cells were cultured in RPMI 1640 (Gibco) medium supplemented with 10% heated‐inactivated bovine growth serum and 1% penicillin/streptomycin (Gibco). THP‐1 cells between 10 and 15 passages were used in this study. All cells were cultured at 37 °C with 5% CO_2_ and passaged twice a week according to the standard protocols.

### Cell Viability Test

3T3 cells were seeded at 100 000 cm^−2^ on the chips. After a pause of 24 h to permit the cells to fully spread, the chips were activated in two modes: continuous mode (input voltage around 2.4 V, input current 5mA, continuously irradiation) and pause mode (input voltage around 2.4 V, input current 5mA, 2 min for one circle: irradiation for 5 s–pause for 115 s–irradiation for 5 s). After the cells were treated several times, they were washed with PBS, and incubated with a live/dead assay (Thermo) in incubator for 30 min. The fluorescence images were then captured by microscopy, and the live/dead ratio was determined through imaging by counting the number of live and dead cells.

### Cell Differentiation and Characterization

Phorbol‐12‐myristate‐13‐acetate (PMA, MCE, 25 ng mL^−1^) was used to induce monocytes differentiation to M0 macrophages. For further polarization, 100 ng mL^−1^ lipopolysaccharide (LPS, Thermo) and 20 ng mL^−1^ interferon‐*γ* (IFN‐*γ*, Thermo) were added to the culture to induce M1 generation. The cells were stimulated to M0 and M1 macrophages for 24 h. Flow cytometry and immunofluorescence staining were used to assess the expression of macrophage‐specific cell surface marker: CD11b for monocyte/macrophage differentiation and CD 80 for M1 macrophage polarization.

### Thrombin Stimulation Study

3T3 cells were grown on the chip surface for overnight and then were washed once and replaced with HEPES buffer (HBSS). After the system was restabilized, various concentrations of thrombin (MCE) were injected into the cell chamber. The signal dynamics were recorded by ammeter.

### Living Cell Calcium Tracking

After the cells were grown on the confocal dish for 24 h, they were washed by PBS and cultured in living cell fluorescence imaging medium (Thermo) with the calcium indicator (Fluo‐3, Ivitrogen, 5 µmol) and pluronic F‐127 (0.02%) in incubator for 1h. They were then washed by fresh culture medium twice and incubated for a further 30 min to allow complete de‐esterification of intracellular acetoxymethyl esters. The living cell fluorescent images were then captured by fluorescence microscope (Zeiss) with the frame rate of 1 side min^−1^.

### Flow Cytometric Measurements

The harvested cells were washed with cold PBS and then the Fc receptor binding sites were blocked by incubating with Human TruStain FcX (422302, Biolegned) on ice for 20 min. The cells were then incubated with either FITC labeled CD 11b (301329, Biolegend) or FITC labeled CD 80 (305206, Biolegend) in darkness for another 30 min. After centrifugation, the cells were washed twice with FACS buffer (PBS containing 2% BSA) and immediately measured by the flow cytometer Novoexpress (Agilent).

### Statistical Analysis

Statistical analyses were performed with GraphPad Prism 8, with statistical significance set at *p* < 0.05 (*p < 0.05, **p < 0.01, ***p < 0.001). Data are represented as mean ± standard deviation (S.D). One‐way analysis of variance (ANOVA) followed by posthoc Tukey's multiple comparisons test was carried out for group differences.

## Conflict of Interest

The authors declare the following competing financial interests: Zhiqin Chu, Yuan Lin, Jixiang Jing, Yong Hou, “Photonic chip for monitoring activities of living cells,” US provisional patent application No. 63/217773.

## Supporting information

Supporting Information

Supplemental Video 1

Supplemental Video 2

Supplemental Video 3

Supplemental Video 4

Supplemental Video 5

## Data Availability

The data that support the findings of this study are available from the corresponding author upon reasonable request.
