# Supplementary material for: A Versatile, Incubator‐Compatible, Monolithic GaN Photonic Chipscope for Label‐Free Monitoring of Live Cell Activities
Source: Adv Sci (Weinh). 2022 Apr 11;9(17):2200910. doi: 10.1002/advs.202200910 (PMC9189681; doi:10.1002/advs.202200910)
Supplement: Supplementary file 1 — Supporting Information [file ADVS-9-2200910-s004.pdf]

ADVANCED SCIENCE
Open Access

# Supporting Information

for *Adv. Sci.*, DOI 10.1002/advs.202200910

A Versatile, Incubator-Compatible, Monolithic GaN Photonic Chipscope for Label-Free Monitoring of Live Cell Activities

*Yong Hou, Jixiang Jing, Yumeng Luo, Feng Xu, Wenyan Xie, Linjie Ma, Xingyu Xia, Qiang Wei, Yuan Lin*, Kwai Hei Li* and Zhiqin Chu**

Supporting Information

# A versatile, incubator-compatible, monolithic GaN photonic chipscope for label-free monitoring of live cell activities

*Yong Hou#, Jixiang Jing#, Yumeng Luo, Feng Xu, Wenyan Xie, Linjie Ma, Xingyu Xia, Qiang Wei, Yuan Lin*, Kwai Hei Li* and Zhiqin Chu**

# These authors have contributed equally to this work.

* Corresponding authors: zqchu@eee.hku.hk, khli@sustech.edu.cn, ylin@hku.hk

**This file includes:**

Figs. S1 to S9
Movies S1 to S5
References

**The images of the photonic chip and the cell chamber**

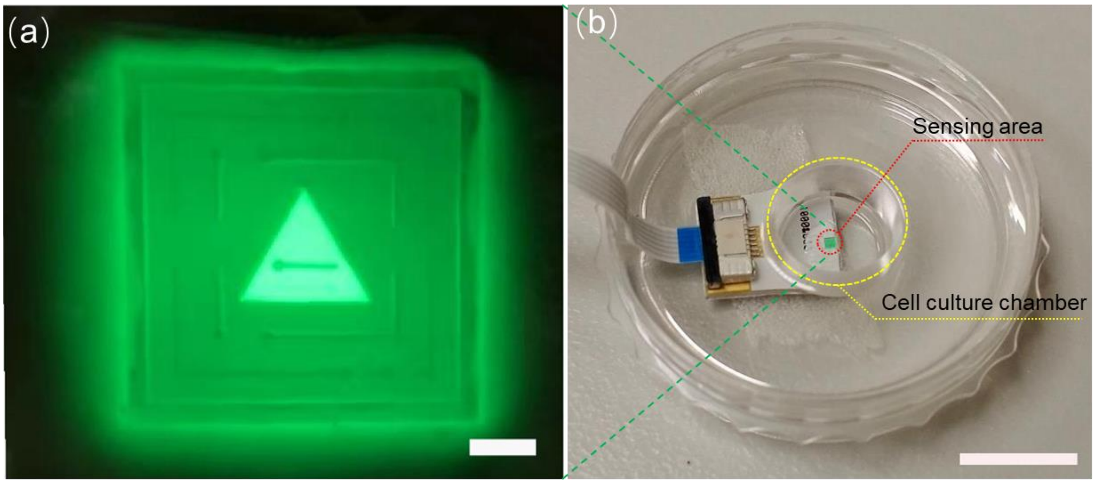


**Fig. S1**. **The images of the GaN chip and the cell chamber integrated with the chip**. (a)The image of the GaN chip that was lightened up. Scale bar indicates 200 μm. (b)The image of the cell chamber on the GaN chip. Scale bar indicates 1 cm.

**Electrical characteristics of the photonic chip**

As shown in Fig. S2a, the I-V curve illustrates that the measured forward voltage of LED is 2.4 V at 10 mA, and the resistance obtained based on the slope of the linear region is 4.95 Ω. Also, the output power of the LED was linearly proportional to the input current. Not surprisingly, as the injecting current in LED increased, the electroluminescence intensity became larger, as shown in Fig. S2b. However, a fixed low input current of 10 mA was used in all our experiments to avoid possible phototoxicity to living cells. Fig. S2c shows the I-V curve of PD under a reverse bias voltage where photocurrent generated by the photodetector was kept at a high level of $10^{-6}$ to $10^{-4}$ A (in contrast to that of ~$10^{-8}$ A without illumination) when the LED injecting current increased from 1 mA to 10 mA. This demonstrates that the measured data possess a high peak signal-to-noise ratio (PSNR).

Additionally, we have determined the chip response time by injecting an electrical pulse into the LED. The LED-converted optical pulse signal is received by a PD connected to a transimpedance amplifier and an oscilloscope. From the measured result shown in **Fig. S3**, our chip can provide fast transition times, with the rise and fall times below 1.5 μs, which was mainly contributed to the fast photon-electron conversion property of the chip device incorporating InGaN/GaN MQWs.

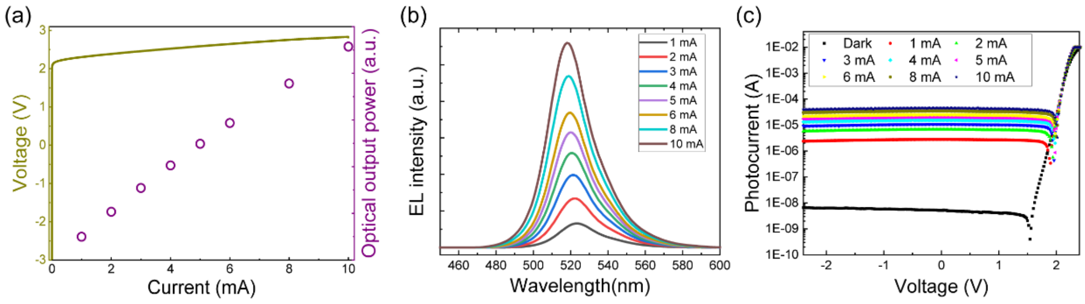


**Fig. S2. Characteristics of the GaN chip.** (a) I−V characteristics of the emitter. The inset shows the L−I characteristics of the emitter. (b) Electroluminescence (EL) spectra of the LED at currents of 1−10 mA measured at room temperature. (c) I−V curves of the detectors. The solid lines and ring-shaped symbols represent the data measured under emitters operating at 10 and 0 mA, respectively.

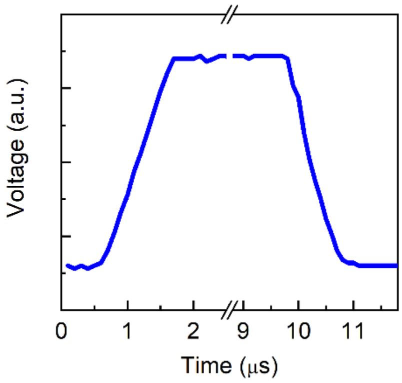


**Fig. S3.** The response of PD when applying an impulse signal on the LED.

**Determination of the vertical sensing range of the GaN chip.**

To determnate the vertical sensing ability of the GaN chip, we conducted the simulation by building a sandwich model with sapphire-intermediate-sample layer to characterize the vertical sensing range of the chip. we defined two possible cases: (1) the vertical separation between the chip and targeted sample layer, and (2) the vertical distance that can be sensed by the chip in the targeted sample layer.

The simulation was conducted by a commercial FEM simulation software, known as COMSOL Multiphysics. Particularly, a sandwich model composed of sapphire/cell/culture medium layers was conducted, and the model construction and solving were in the 2D Wave Optics module. Plane-wave with different incident angles and one-unit cell by applied periodic boundary conditions were performed in this work. The refractive indexes of the sapphire and culture medium are fixed at 1.78 and 1.34, respectively, while the refractive index of the cells is set to a range of 1.35-1.37.[1]

Case 1: We supposed the targeted sensing layer as the monolayer cells. When the intermediate between the chip and sensing layer is air, the total reflectance (internal reflectance and scattering) responds to a limited distance ranging from 0 nm to 300 nm (**Fig. S4a and b**). When the intermediate medium changes to water, the vertical responsive distance is 0-500 nm (**Fig. S4c and d**). Therefore, the theoretical maximum vertical sensing range will be around 300 nm and 500 nm for air and water, respectively.

Case 2: The degree of reflectance is governed by the refractive index difference at the interface. During the cell detection process, there exist two interfaces (sapphire/cell and cell/culture medium) above the chip, as illustrated in **Fig. S5a.** When the incident angle exceeds the critical angle ($\theta c$=~50°) at the sapphire/cell interface, the light undergoes total internal reflection, as shown in **Fig. S5b**. Only light rays with an incident angle less than the critical angle will partially enter the cell. However, the weak refractive index contrast at the culture medium/cell interface leads to a large critical angle of >78°, as illustrated in **Fig. S5c**. Moreover, the culture medium/cell interface provides very weak reflectance, and the amount of light that can be reflected is highly limited.

During measurements, it is expected that the lateral spreading of the cell across the chip surface can increase the amount of reflected light at the culture medium/cell interface. However, the photocurrent magnitude is found to decrease monotonically over time, implying that reflected light from this part is negligible. As such, light undergoing total internal reflection at the sapphire/cell interface remains the dominant part.

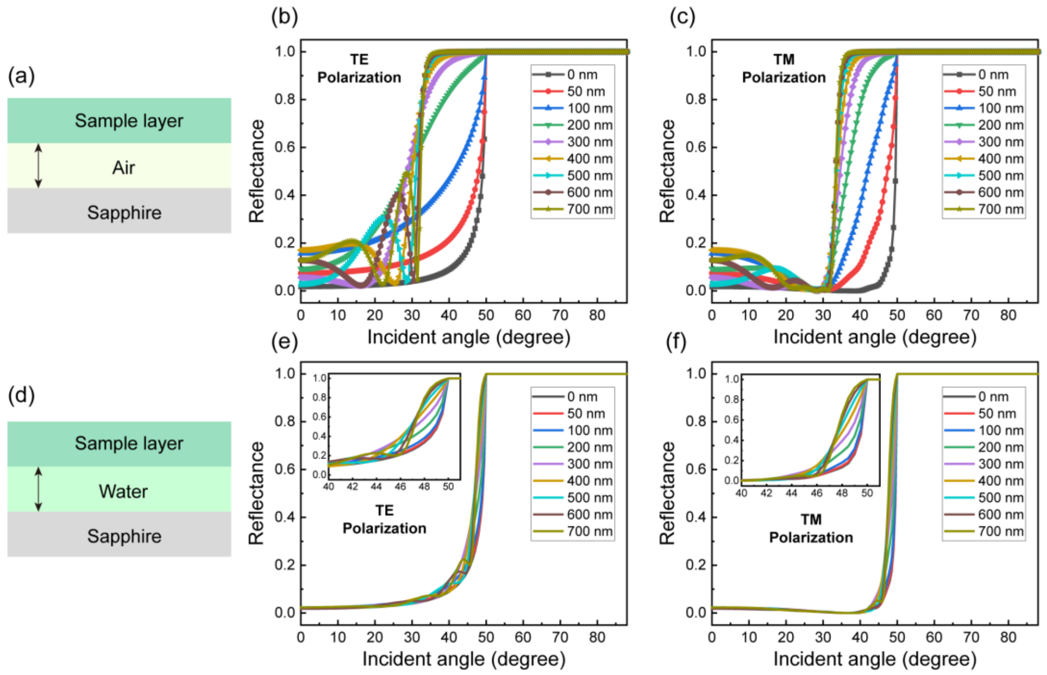


**Fig.S4.** (a) Schematic of sensing model for different thickness of air layer, and corresponding calculated (b) TE- and (c) TM- polarized reflectance. (d) Schematic of sensing model for different thickness of water layer, and corresponding calculated (e) TE- and (f) TM- polarized reflectance. The inset is the local enlarged image.

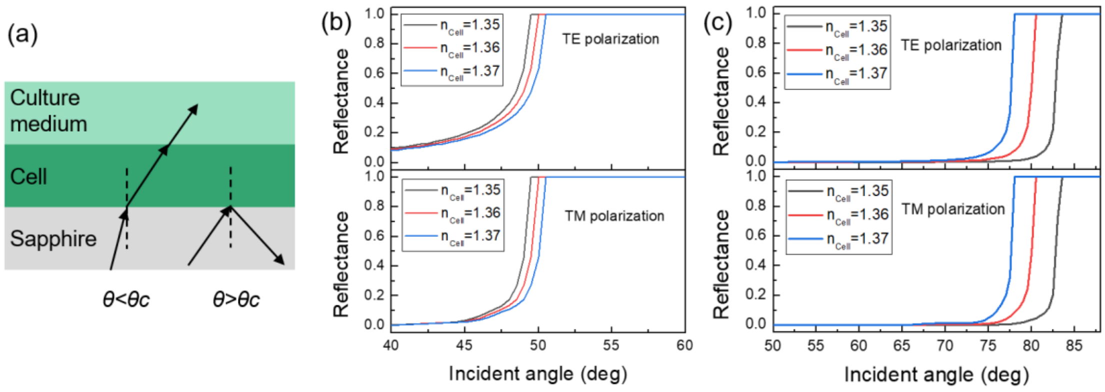


**Fig. S5**. (a) Schematic of light propagation at different interfaces. Calculated TE and TM polarized reflectance at (b) sapphire/cell and (c) cell/culture medium interfaces, respectively.

**Phototoxicity study of the GaN chip.**

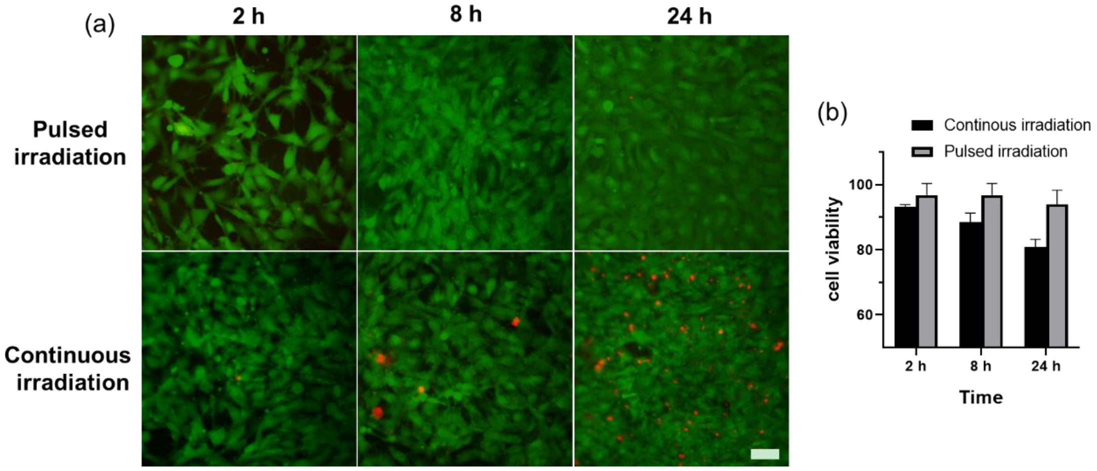


**Fig. S6**. **The cell viability study of the GaN chip for the cells.** Input voltage 2.4 V, input current 10 mA. Pulsed irradiation: 2 min for one circle: irradiation for 5s -pause for 115s - irradiation for 5s. (A) The live/dead staining of the treated cells on the chip. Green color indicates the live cells staining with calcein-AM. Red color indicates the dead cells staining with ethidium homodimer-1. Scale bar indicates 100 µm. (b) The cell viability was determined by counting the live/dead cells ratio. Data are presented as the mean ± SD, (N= 4-5).

**Fabrication of the cell adhesion-resistance surface on the GaN chip**

To establish a cell adhesion resistance surface on the GaN chip, a monolayer polymer coating based on liner polyglycerol (LPG) is employed in this work, which has been proved capable of providing effective antifouling properties in various substrates.[2] The fabrication of the antifouling polymer layer on the device is via two steps: 1) a hydrophobic layer is formed on the sapphire surface of the device by sialyation; 2) amphiphilic block copolymers benzophenone functionalized liner polyglycerol (LPG-BPh) self-assemble on the alkyl-functionalized substrates through the hydrophobic-hydrophobic interaction between the hydrophobic domain (BPh) of the polymer and hydrophobic base alkyl layer.[3] Then, the polymers were covalently bonded on the alkylated sapphire by the UV initiated "C-H" photo-crosslinking between BPh groups and neighboring "C-H". The thickness of the monolayer coatings is about 3.5 nm.[3] Figures S3 shows the surface morphologies of the sapphire face of the device without and with the LPG coating. The island-like pattern from Figure S3b corresponds to the surface feature of monolayer polymer brush coating.[4] In addition, no significant changes in the roughness can be observed after the surface engineering (bare chip: $R_a$ = 3.46 nm, LPG @chip: $R_a$ = 3.15 nm).

*Hydrophobic layer establishment on the chip surface:* The cleaned chips were actived by the surface plasma, and then were immersed in ethanol solution containing 30% v/v acetic acid and

trimethoxy-(octyl)silane (0.5 M, for octyl substrate) in a big-neck flask. The flask was placed at room temperature for 1 day. After that, the slides were thoroughly rinsed by ethanol and dried with $N_2$ stream.

*Antifouling coating preparation:* The antifouling coating was prepared via a simaple one step dip-coating method. The cleaned octyl substrates were dip into a solution of 1 mg/mL LPG-BPh in Milli-Q water at room temperature for overnight. After that, the coated chip were thoroughly rinsed with water and dried by $N_2$ stream.

*Surface characterization:* AFM data was got by a NanoWizard 4XP scanning probe microscope (Bruker, USA) in air. The images were got from AC Mode with commercially available AFM cantilever tips (TESP-V2, Bruker) with a spring constant 42 N/m.

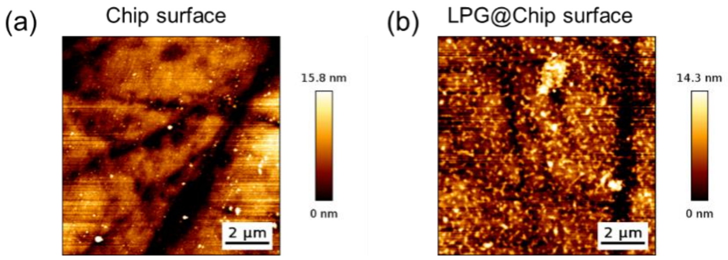


**Fig. S7. Surface morphology of the GaN chip illustrated by the AFM.** (a) Bare GaN chip surface. (b) LPG@GaN chip.

**Living cell calcium tracking**

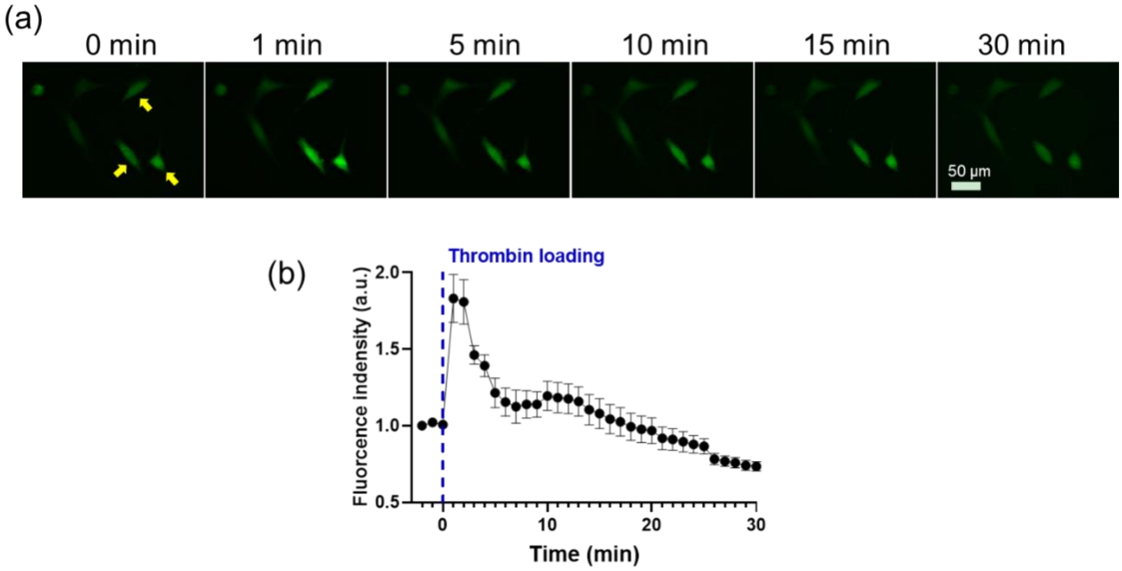


**Fig. S8. Living cell calcium tracking after the loading of low dose of thrombin**. (a) The time laps fluorescent images of the calcium (green) in living cells (thrombin 2U/mL). (b) The calcium fluorescence intensity was quantified. Data are presented as the mean ± SD, (N=3).

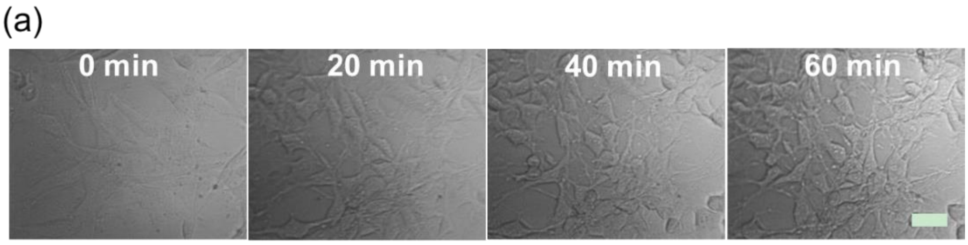


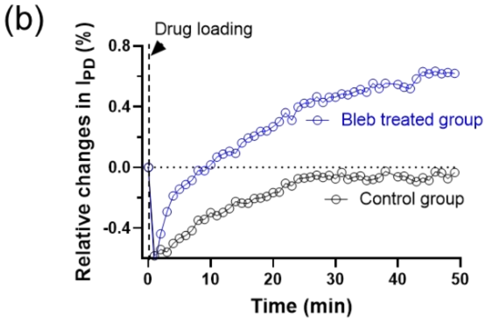


**Fig. S9. Label-free monitoring of cell dynamics after treated with blebbistatin.** (a) Representative images of 3T3 cells treated with blebbistatin (50 μM) at different time points. Scale bar indicates 50 μm. (b) The relative of optical current as a function of time during the inhibitor treatment.

**Table S1:The summary of the technologies in the application of label-free living cell activities sensing**

| | Sensitivity | Vertical sensing range | Size | Incubator compatibility | Integrability and miniaturization | Complexity |
|---|---|---|---|---|---|---|
| Impedance sensor | $10^4$–$10^{-4}$ μmol/L[5] | 0 | Small | Yes | Yes | Low (electrode pattern +readout circuit) |
| Resonant waveguide grating biosensor (RWG) | $10^2$–$10^4$ degree/RIU[6] | ~100 nm | N/A | No | No | High (Laser +spectrometer+ prism) |
| Surface plasmon resonance (SPR) | $0.4\times10^2$-$10^3$ degree/RIU[6] | ~100 nm | N/A | No | No | High (Laser +spectrometer+ prism) |
| Monolithic GaN chip | 149216 nA/ RIU* | ~500 nm* | ~ mm | Yes | Yes | Low （GaN chip+ ammeter） |

*The data was collected from this work. The vertical sensing range was calculated by the COMSOL Multiphysics software, herein, the range represent the vertical separated distance between the chip and sample layer when the intermediate is water, referred to Fig. S4.

**Movie S1.**
Monitoring the 3T3 cells initial attachment and spreading on the adhesive surface.

**Movie S2.**
Monitoring the 3T3 cells initial attachment and spreading on the non-adhesive surface.

**Movie S3.**
Monitoring the intracellular calcium signals dynamics of 3T3 cell after loading of low dose of thrombin.

**Movie S4.**
Monitoring the monocytes differentiate to macrophage (M0).

**Movie S5.**
Monitoring the macrophage (M0) differentiate to M1.

**References:**

[1] F. Lanni, A. S. Waggoner, D. L. Taylor, *J. Cell Biol*. **1985**, 100, 1091.

[2] L. Yu, C. Cheng, Q. Ran, C. Schlaich, P. L. M. Noeske, W. Li, Q. Wei, R. Haag, *ACS Appl. Mater. Interfaces* **2017**, 9, 6624.

[3] L. Yu, Y. Hou, W. Xie, J. L. C. Camacho, C. Cheng, A. Holle, J. Young, B. Trappmann, W. Zhao, M. F. Melzig, E. A. Cavalcanti-Adam, C. Zhao, J. P. Spatz, Q. Wei, R. Haag, *Adv. Mater*. **2020**, 32, 2002566.

[4] L. Yu, Y. Hou, C. Cheng, C. Schlaich, P. L. M. Noeske, Q. Wei, R. Haag, *ACS Appl. Mater. Interfaces* **2017**, 9, 44281.

[5] D. Grieshaber, R. MacKenzie, J. Vörös, E. Reimhult, *Sensors* **2008**, 8, 1400.

[6] V. Yesudasu, H. S. Pradhan, R. J. Pandya, *Heliyon* **2021**, 7, e06321.
